# A Multi-million Mammography Image Dataset and Population-Based Screening Cohort for the Training and Evaluation of Deep Neural Networks—the Cohort of Screen-Aged Women (CSAW)

**DOI:** 10.1007/s10278-019-00278-0

**Published:** 2019-09-13

**Authors:** Karin Dembrower, Peter Lindholm, Fredrik Strand

**Affiliations:** 1 Department of Physiology and Pharmacology, Karolinska Institutet, Solna, Sweden; 2 Breast Radiology Department of Radiology, Capio Sankt Görans Hospital, Stockholm, Sweden; 3 Thoracic Radiology, Imaging and Physiology, Karolinska University Hospital, Solna, Sweden; 4 Department of Pathology and Oncology, Karolinska Institutet, Solna, Sweden; 5 Breast Radiology, Karolinska University Hospital, Solna, Sweden

**Keywords:** Dataset, Machine learning, Breast cancer, Screening, Mammography

## Abstract

For AI researchers, access to a large and well-curated dataset is crucial. Working in the field of breast radiology, our aim was to develop a high-quality platform that can be used for evaluation of networks aiming to predict breast cancer risk, estimate mammographic sensitivity, and detect tumors. Our dataset, Cohort of Screen-Aged Women (CSAW), is a population-based cohort of all women 40 to 74 years of age invited to screening in the Stockholm region, Sweden, between 2008 and 2015. All women were invited to mammography screening every 18 to 24 months free of charge. Images were collected from the PACS of the three breast centers that completely cover the region. DICOM metadata were collected together with the images. Screening decisions and clinical outcome data were collected by linkage to the regional cancer center registers. Incident cancer cases, from one center, were pixel-level annotated by a radiologist. A separate subset for efficient evaluation of external networks was defined for the uptake area of one center. The collection and use of the dataset for the purpose of AI research has been approved by the Ethical Review Board. CSAW included 499,807 women invited to screening between 2008 and 2015 with a total of 1,182,733 completed screening examinations. Around 2 million mammography images have currently been collected, including all images for women who developed breast cancer. There were 10,582 women diagnosed with breast cancer; for 8463, it was their first breast cancer. Clinical data include biopsy-verified breast cancer diagnoses, histological origin, tumor size, lymph node status, Elston grade, and receptor status. One thousand eight hundred ninety-one images of 898 women had tumors pixel level annotated including any tumor signs in the prior negative screening mammogram. Our dataset has already been used for evaluation by several research groups. We have defined a high-volume platform for training and evaluation of deep neural networks in the domain of mammographic imaging.

**Electronic supplementary material:**

The online version of this article (10.1007/s10278-019-00278-0) contains supplementary material, which is available to authorized users.

## Background

Developing deep neural networks in the field of radiology is the focus of many research groups [1, 2]. Having a refined network architecture is important, while access to a large and well-curated dataset is crucial. There are a few large datasets available for deep learning research. NIH has released a data set of 100,000 chest X-rays from 30,000 patients [3]. The Stanford dataset CheXpert features 224,316 chest X-rays and radiology reports from 65,240 patients [4]. A group from the Geisinger Health system in the USA has curated a dataset of 40,367 3D head CT studies and trained a deep learning system for detecting brain hemorrhage [5]. For breast imaging, there are a few publicly available mammographic datasets, such as Mammographic Image Analysis Society Minimammographic Database (mini-MIAS) and Digital Database for Screening Mammography (DDSM). They have a relatively small number of examinations: in mini-MIAS, there are 322 digitized films, and in DDSM there are 2500 examinations. An advantage of the DDSM dataset is the inclusion of pixel-level annotations. However, the number of cases is very small in relation to what is required for deep neural networks, and the datasets are not representative of screening populations.

Sweden has a long tradition of breast cancer screening. National screening programs started in 1986 [6] and large mortality studies have shown that breast cancer mortality decreased approximately 30% after introducing screening [7, 8]. In Sweden, all women between 40 and 74 years are invited for breast cancer screening every 18 to 24 months. The attendance rate is around 80% [9]. Around 3% of all screened women are recalled for additional imaging [10] and around 0.5% get a diagnosis of breast cancer [11]. For women attending screening, around 70% of all breast cancers are screen-detected [12] and the remaining 30% are interval cancers. One aim of AI systems, in breast imaging, is to improve early detection by computer-aided detection (CAD) of tumors that would otherwise have resulted in false negative screenings. Another aim of AI networks could also be to predict which women would benefit the most from more sensitive, but expensive and time-consuming, supplemental screening modalities such as magnetic resonance imaging (MRI). Traditional image-based risk prediction models have mainly utilized a single measure of breast density [13]. By using deep learning techniques more information than density can potentially be extracted from the mammograms.

Working in the field of breast radiology, our aim has been to develop a high-quality platform for training and testing of AI networks for screening mammography. The well-established breast cancer screening program in Sweden, with national guidelines and government-run cancer databases, provided an excellent opportunity to create a large dataset [14]. Linkage between registers and follow-up over time was facilitated by the Swedish personal identification numbers.

## Method

### CSAW Cohort

Our dataset Cohort of Screen-Aged Women (CSAW) is a complete population-based cohort of women 40 to 74 years of age invited to screening in the Stockholm region, Sweden, between 2008 and 2015. All women were invited to mammography screening free of charge every 18 to 24 months. The regional cancer center provided personal identification numbers for all women that fulfilled the inclusion criteria. The identification numbers were linked to the breast cancer quality register to extract the following information: time variables (of each visit, of cancer diagnosis, and of death if any), diagnostic variables (tumor location, treating clinic, clinical/screening detection, detection method, invasiveness), surgical variables (surgical method, reoperation status, axilla surgery), and data for tumor, node, and metastasis (TNM) classification. Tumor biology variables whereof the most important are tumor receptor status (progesterone, estrogen, and herceptin), histological origin (ductal, lobular, medullary, phyllodes), Elston grade (grade 1 to 3), and lymph node status. All diagnoses of breast cancer were biopsy verified. Molecular subtypes were defined using receptor proxies: Luminal A for cancers that were positive for both estrogen and progesterone receptors and Her2 receptor negative; Luminal B for cancers that were positive for either estrogen or progesterone receptor and Her2 receptor negative; Her2 overexpressing for cancers that were Her2 positive; and Basal for cancers that were estrogen, progesterone, and Her2 receptor negative. The personal identification numbers were also linked to the radiological image repository to extract all digital mammograms from the PACS of the three breast centers that completely cover the region: Karolinska University Hospital, Sankt Goran Hospital, and Southern General Hospital. Incident cancer cases were pixel level annotated by a radiologist. DICOM metadata, including equipment manufacturer, compressed breast thickness, and exposure information, were collected together with the images. Screening decisions and clinical outcome data were collected by linkage to the regional cancer center registers. All mammography screening examinations had been assessed by two radiologists independently and the following screening decision data were collected: flagging of potential pathology by none, one or both radiologists, and the final recall decision after consensus discussion. The images and corresponding data were then anonymized and stored on a local off-line server. A nested case-control subset for efficient evaluation of external networks was defined as described below. The collection and use of the dataset for the purpose of AI research has been approved by the regional Ethical Review Board (ERB). The ERB waived the requirement for informed consent, which meant that the cohort should include all women without bias. Image retrieval was additionally approved by each head of radiology department.

### Image Annotation

Once the images and data had been stored, we selected women who had been diagnosed with breast cancer and retrieved their images from the time of diagnosis, if available, and from prior screening. For mammographically visible tumors, we used an in-house tool to free-hand annotate regions of interest (ROI) on a pixel level. We also annotated any tumor signs that we could identify in the prior, supposedly negative, screening mammogram. If there were no tumor signs in the prior mammogram, we identified the pixel coordinates of the corresponding location of where the tumor subsequently arose by comparing with the mammogram from time of diagnosis. Some of the mammograms had no visible tumor sign, and no annotation was made in those images. Images were assessed on a high-resolution diagnostic grade display. The in-house annotation tool was developed using the MATLAB software (MathWorks). Image annotation was limited to women diagnosed at the Karolinska University Hospital.

### CSAW Case-Control Subset

Based on women attending screening at the Karolinska University Hospital, we have defined a separate case-control subset containing all data for all breast cancer cases and 10,000 randomly selected healthy controls. The purpose of the case-control subset is to make evaluation more efficient by not having to process an unnecessary amount of healthy controls while preserving the representability of the CSAW screening cohort in which it is nested. All images were acquired on Hologic mammography equipment. The subset includes the breast cancer cases for which the pixel-level annotations were made. Subset data were transferred and stored on local SSD hard drives on a separate evaluation workstation equipped with Ubuntu operating system, 20 cores, 32 GB RAM, Gigabyte GeForce GTX 1080 Ti graphics card, 256 GB SSD drive for operating system, and application use as well as an additional 10 TB external hard drive. Installed software includes TeamViewer, Docker, and Virtual Box. When external parties remotely access the evaluation workstation, the SSD image drives are temporarily detached to protect patient integrity and data safety. The system set-up is intended for evaluation purposes and would not be recommended for efficient training of deep neural networks.

## Results

**Fig. 1:**
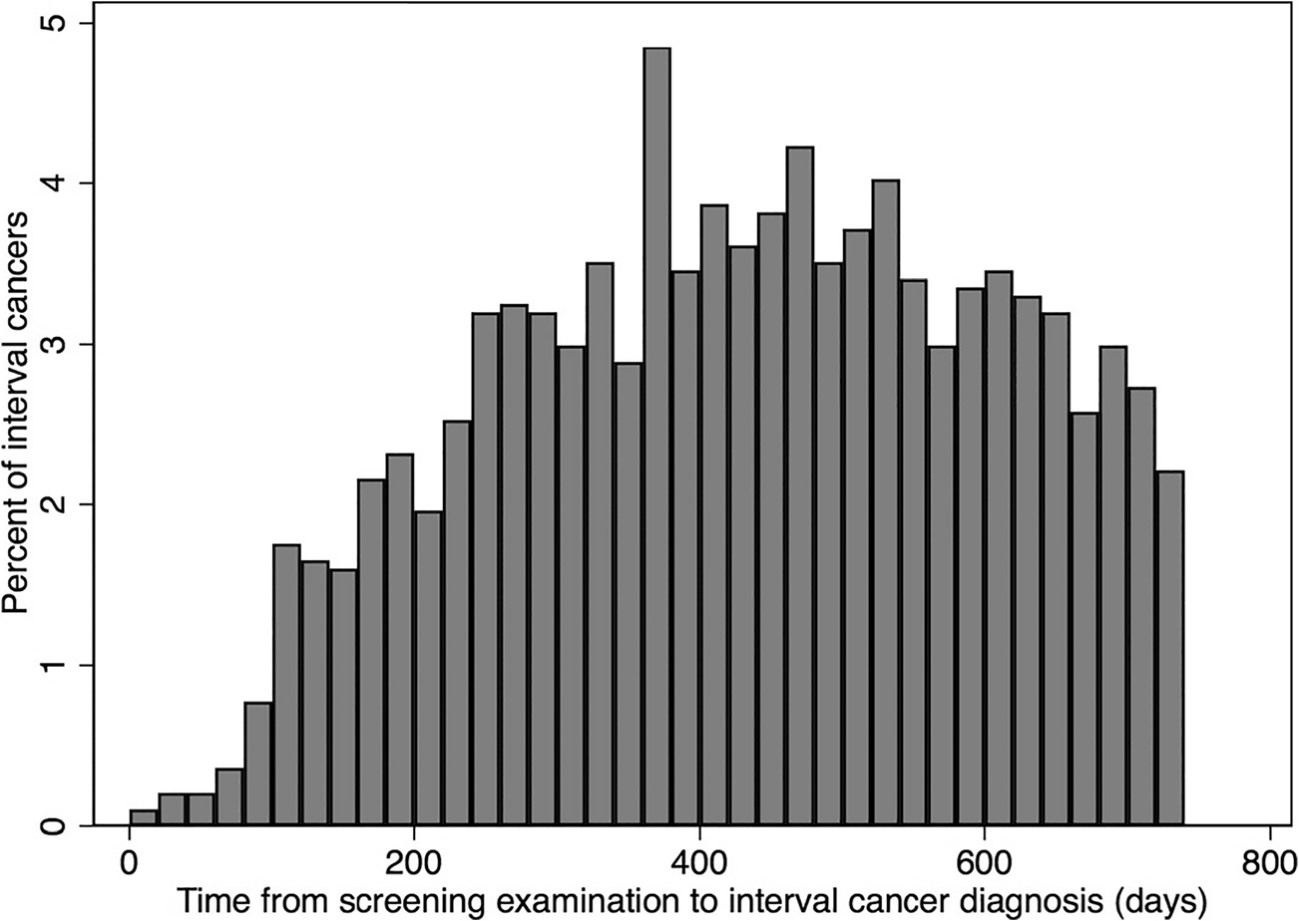
Time from each screening examination to time of diagnosis for interval cancers (*n* = 1938), i.e., cancers detected during the 2-year interval after a negative screening examination. The bar chart shows that immediately after a negative screening the rate of clinically detected interval cancer is low, and then gradually increases until around 400 days afterwards

**Fig. 2:**
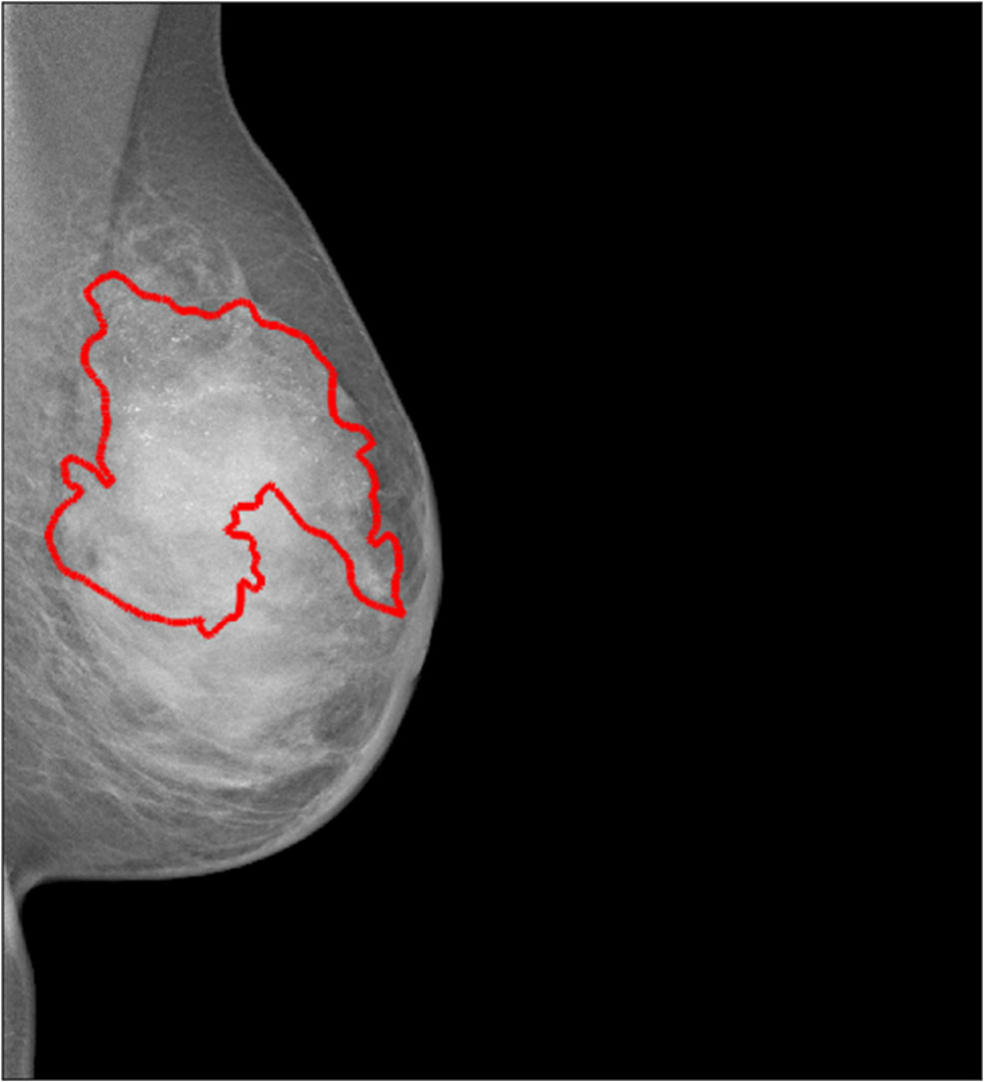
Example of pixel-level annotation of an in situ cancer which is characterized by multiple calcifications

In total, 499,807 women were included in the CSAW cohort on the basis of a total of 1,688,216 invitations to screening between 2008 and 2015. There were 2119 women with a prior history of breast cancer or diagnosed at an age outside the screening range. After excluding these women, there were 8463 women diagnosed with their first incident breast cancer (Table 1). The average age was 53.2 years (SD 10.1) overall and for healthy women and 57.8 (SD 9.3) for women diagnosed with breast cancer (*p* < 0.001). As a result of 1,688,216 invitations to screening, there was a total of 1,182,733 (70%) completed screening examinations (Table 2). Each examination consisted of four images, two views of each breast. Most women had 3 to 4 screening rounds during the study time period. There were 4703 screen-detected cancers (SDC) and 1938 interval cancers (IC) (Table 3). The proportion of IC of the sum of IC and SDC was 29%. The time from prior negative screening to the time of IC diagnosis is shown in Fig. 1. The most common invasive cancer was ductal (67%, *n* = 5632) and the second most common was lobular (11%, *n* = 922). The median sizes for invasive-only and in situ cancers were 15 mm and 21 mm, respectively. The total number of images in the cohort was more than 4 million. As of today, around 2 million images, including all breast cancer cases, have been transferred to the locally stored dataset. Pixel-level annotations were made in 1891 mammograms of 898 women (Table 4, Fig. 2).

**Table 1:** Description of all women in the CSAW study population

Cancer status	Women	%
Total	499,807	100.0%
Healthy (at least until Dec. 31, 2015)	489,225	97.5%
Diagnosed with cancer	10,582	2.1%
Prior cancer or age outside screening range	2119	0.4%
Incident cancer (2008 to 2015)	8463	1.7%

**Table 2:** Mammography screening examinations

	Invitation to screening		Completed examination	
	*n*	%	*n*	%
Karolinska University Hospital	278,996	17%	198,820	17%
Sankt G8ran Hospital	668,366	40%	454,341	38%
Southern General Hospital	482,883	29%	340,866	29%
Danderyd Hospital	257,717	15%	188,527	16%
Other	254	< 1%	179	< 1%
Total	1,688,216	100%	1,182,733	100%

Each screening examination contains four images, two of each breast

**Table 3:** Description of incident breast cancer cases

Women with prior cancer were not included		
Parameter	*n*	%
Age at breast cancer diagnosis (years)		
40 to 49	1927	23%
50 to 59	2379	28%
60 to 69	3227	38%
70+	930	11%
Missing information	0	0%
Detection mode		
Screen-detected cancer	4703	56%
Interval cancer (2-year interval)	1938	23%
Overdue > 3 years	393	5%
Never-screened	566	7%
Indeterminate detection mode	863	10%
Missing information	0	0%
Tumor size		
0–5 mm	278	3%
6–10 mm	1263	15%
11–19 mm	3014	36%
20+ mm	2476	29%
Missing information	1432	17%
Invasiveness		
Invasive only	3130	37%
In situ only	1080	13%
Mixed invasive and in situ	4117	49%
Missing information	136	2%
Lymph node status		
Negative, no metastasis	7572	89%
Positive, metastasis detected	876	10%
Missing information	15	0%
Histology		
Ductal only	5632	67%
Lobular only	922	11%
Medullary	43	1%
Mixed or other	623	7%
Missing information or in situ cancer	1243	15%
Receptor status		
Estrogen receptor positive	6599	78%
Estrogen receptor negative	911	11%
Missing information	953	11%
Progesterone receptor positive	5527	65%
Progesterone receptor negative	1855	22%
Missing information	1081	13%
HER2 receptor amplified	902	11%
HER2 receptor negative	5463	65%
Missing information	2098	25%

**Table 4:** Pixel-level annotations of tumors at diagnosis and prior screening

Annotation type		Women	Images
Tumor annotated, total		898	1891
Tumor annotated, at diagnosis		896	1761
Tumor annotated, at prior screening		72	139
Location defined, no tumor, at prior screening		177	335
Annotation measure	25%-tile	Median	75%-tile
Area (pixels^2^), at diagnosis (*n* = 1741)	15,780	32,906	72,383
Area (pixels^2^), at prior screening (*n* = 130)	3573	7027	11,773
Major axis (pixels), at diagnosis (*n* = 1741)	177	258	396
Major axis (pixels), at prior screening (*n* = 130)	83	118	163

For the case-control subset which is available for evaluation of external networks, we included women from Karolinska University Hospital. Additional cases and controls from the other two breast centers in Stockholm will be added shortly. All images for women diagnosed with their first breast cancer (*n* = 1303) and all images for 10,000 randomly selected controls were included. All images in the case-control subset were acquired on Hologic® mammography systems. The case-control subset is currently used in studies evaluating the performance of AI CAD deep neural networks and for comparing network performance with radiologist performance. The following parameters are examples of what can be evaluated: abnormal interpretation rate, recall rate, cancer detection rate, false negative rate, false positive rate, sensitivity, specificity, and AUC. We can also estimate hazard ratio and odds ratio for the association between deep neural network predictions and breast cancer within a follow-up time period, for all breast cancers and for screen-detected and interval cancers separately. These performance parameters can then be further evaluated based on any of the clinical cancer data collected such as tumor size, histological origin, or molecular subtype (Supplemental Table S1), or by image acquisition parameters (Supplemental Table S2).

## Discussion

We have curated a large cohort of women, CSAW, based on invitation to screening in a geographically defined area. Compared with other public mammography datasets with hundreds to thousands of images, our dataset contains millions of images. The CSAW dataset has been used as evaluation data in the Digital Mammography DREAM Challenge and in our own research [2, 15–17]. In addition, there is an on-going evaluation work for four external research groups.

We have linked each woman to pathological mammograms and normal mammograms. The dataset contains information about cancer diagnosis, staging, and tumor characteristics as well as surgical characteristics, radiological assessments, and image acquisition metadata. In our data, we observed a higher age for women who were diagnosed with breast cancer compared with those who remained healthy, which agrees with prior studies [18]. We found that nearly 30% of cancers were not screen-detected but diagnosed clinically during the interval between screening examinations, in line with prior numbers in a pooled analysis of six European countries [19]. There is high reliability of the diagnoses since more than 99% are biopsy verified and underreporting to the cancer registry is around 1.1 to 1.6% [20]. Many research questions regarding breast screening and cancer diagnoses can be addressed in the context of deep neural networks by using the CSAW dataset. We have listed a few potential application areas below:

1. **Developing risk prediction networks**. By training a network on healthy mammograms and mammograms from women who later developed cancer, a risk prediction score can be calculated for each woman. The score can eventually be used as a tool to select high-risk women versus low-risk women. Upon this, more individualized screening schemes can be developed.
2. **Developing tumor detection networks.** By training a network on healthy mammograms and on mammograms containing tumor(s), the network can discriminate between healthy and pathologic mammograms. The tumor detection network can eventually be used in many other environments such as acting as a single reader which is attractive in the light of a lack of breast radiologists today. Tumor detection networks can also act as an assessment of the radiologists’ capability of assessing mammograms and the false negative recall rate.
3. **Developing sensitivity assessment networks.** A network can be trained on “normal” images of women that later developed interval cancer, i.e., the negative screening mammogram before the interval cancer was detected. Thus, the network could potentially learn to discriminate between mammographic appearances representing high and low sensitivity.
4. **Evaluating and validating third-party networks.** The case-control subset of CSAW is an enriched representative dataset of mammograms based on a full screening population. In expectation of a large number of competing AI networks, there is an increasing need for robust external evaluation of them.
5. **Interactive education and continuous training system.** Images with and without the annotated cancers can potentially be used as interactive training cases in educational software. If available, deep learning predictions for tumor detection and for mammographic sensitivity can be used to assess the difficulty level of each case. The most appropriate cases can then be selected for each trainee’s proficiency level. By leveraging the clinical cancer data, training cases can be enriched with, e.g., lobular cancers for a trainee who performs relatively worse for that subtype.

A strength of our dataset is that all women that were invited to screening are included, without exclusions. Another strength is the large number of diagnosed women, and the large number of clinical cancer data and image acquisition parameters that are available for subgroup analysis and adjustments. Finally, the free-hand pixel-level annotations by an experienced breast radiologist make precise locational comparisons possible. A potential limitation of our dataset is that even though it is large, it might still be too small for any given training task. We have previously demonstrated that a limited sample from the dataset was enough to develop a deep neural network that achieved a similar, or better, performance to breast density in breast cancer risk prediction [16]. The current case-control dataset was composed of images from one vendor only, which restricts the evaluation. Going forward, we plan to add breast cancer cases from the other two breast centers in Stockholm, which will include images acquired on equipment from other vendors.

## Conclusion

For around 500,000 women, we have collected screening assessment data, clinical cancer data, and mammograms of all 10,582 women who were diagnosed with breast cancer as well as a random selection of mammograms of women who remained healthy. CSAW allows training of deep neural networks for diverse applications. An enriched case-control dataset on a separate computer is available for external researchers providing that applicable rules and regulations are followed. To gain access, please communicate directly with the last author of this paper (first name.last name@ki.se).

## Electronic Supplementary Material

ESM 1 (DOCX 32 kb)
