# Supplementary material for: A Multi-million Mammography Image Dataset and Population-Based Screening Cohort for the Training and Evaluation of Deep Neural Networks—the Cohort of Screen-Aged Women (CSAW)
Source: J Digit Imaging. 2019 Sep 13;33(2):408–13. doi: 10.1007/s10278-019-00278-0 (PMC7165146; doi:10.1007/s10278-019-00278-0)
Supplement: Supplementary file 1 — (DOCX 32 kb) [file 10278_2019_278_MOESM1_ESM.docx]

**Supplemental Table S1.** Image acquisition parameters - available for each image.

Study date

Age

Laterality

View position

Body part thickness

Breast implant present

Detector element spacing

Entranced dose

Exposure

Exposure time

Imager pixel spacing

kVp

Manufacturer

Manufacturer's model name

Organ dose

Rows

Window center

Window width

X-ray tube current

**Supplemental Table S2.** Clinical cancer data - available for each diagnosed cancer.

Number of prior breast cancer diagnoses

Date at diagnosis

Laterality

Age at diagnosis

T class

N class

M class

Pre-operative treatment

Invasiveness

Histology

Multifocality

Size of invasive component

Size of in situ component

Number of lymph node metastases

Percent of estrogen receptor (ER) expression

Binary status of ER expression

Percent of progesterone receptor (PR) expression

Binary status of PR expression

Percent of human epidermal growth factor receptor 2 (HER2) expression

Binary status of HER2 expression
